# Ac2-26 Mimetic Peptide of Annexin A1 Inhibits Local and Systemic Inflammatory Processes Induced by *Bothrops moojeni* Venom and the Lys-49 Phospholipase A_2_ in a Rat Model

**DOI:** 10.1371/journal.pone.0130803

**Published:** 2015-07-06

**Authors:** Bruna Stuqui, Marina de Paula-Silva, Carla Patrícia Carlos, Anwar Ullah, Raghuvir Krishnaswamy Arni, Cristiane Damas Gil, Sonia Maria Oliani

**Affiliations:** 1 Laboratory of Immunomorphology, Department of Biology, São Paulo State University (UNESP), São José do Rio Preto, São Paulo, Brazil; 2 Multiuser Center for Biomolecular Innovation, Department of Physics, São Paulo State University (UNESP), São José do Rio Preto, São Paulo, Brazil; 3 Department of Morphology and Genetics, São Paulo Federal University (UNIFESP), São Paulo, Brazil; Universidad de Costa Rica, COSTA RICA

## Abstract

Annexin A1 (AnxA1) is an endogenous glucocorticoid regulated protein that modulates anti-inflammatory process and its therapeutic potential has recently been recognized in a range of systemic inflammatory disorders. The effect of the N-terminal peptide Ac2-26 of AnxA1 on the toxic activities of *Bothrops moojeni* crude venom (CV) and its myotoxin II (MjTX-II) were evaluated using a peritonitis rat model. Peritonitis was induced by the intraperitoneal injection of either CV or MjTX-II, a Lys-49 phospholipase A_2_. Fifteen minutes after the injection, the rats were treated with either Ac2-26 or PBS. Four hours later, the CV and MjTX-II-induced peritonitis were characterized by neutrophilia (in the peritoneal exudate, blood and mesentery) and increased number of mesenteric degranulated mast cells and macrophages. At 24 hours post-injection, the local inflammatory response was attenuated in the CV-induced peritonitis while the MjTX-II group exhibited neutrophilia (peritoneal exudates and blood). Ac2-26 treatment prevented the influx of neutrophils in MjTX-II–induced peritonitis and diminished the proportion of mesenteric degranulated mast cells and macrophages in CV-induced peritonitis. Additionally, CV and MjTX-II promoted increased levels of IL-1β and IL-6 in the peritoneal exudates which were significantly reduced after Ac2-26 treatment. At 4 and 24 hours, the endogenous expression of AnxA1 was upregulated in the mesenteric neutrophils (CV and MjTX-II groups) and mast cells (CV group). In the kidneys, CV and MjTX-II administrations were associated with an increased number of macrophages and morphological alterations in the juxtamedullary nephrons in proximal and distal tubules. Ac2-26 promoted significant recovery of the juxtamedullary structures, decreased the number of macrophages and diminished the AnxA1 in epithelial cells from distal tubules and renal capsules. Our results show that Ac2-26 treatment significantly attenuates local and systemic inflammatory processes and indicate this peptide as a potential target for the development of new therapeutic strategies for the snakebite envenomation treatment.

## Introduction

Ophidic accidents constitute a serious and neglected public health problem in tropical countries [1]. In Brazil, there were 25,302 cases of ophidic envenomation reported in 2013, primarily caused by snakes belonging to the genus *Bothrops*, [2] of which 0.5% were lethal.

*Bothrops* snake venoms induce a pathophysiological condition characterized by local and systemic effects [3, 4]. The local effects include pain, edema, local hemorrhage, inflammation and bruising [5–7] and generally result in tissue necrosis [3]. The systemic effects result in clotting, cardiovascular and renal alterations, hypovolemic shock and bleeding at sites distant from the bite [4]. Some of the primary components responsible for the pathophysiological effects of bothropic venoms are the phospholipases A_2_ (PLA_2_s), which are a family of lipolytic enzymes that play key roles in several cellular processes by regulating the release of arachidonic acid and lysophospholipids from cell membrane phospholipids [8]. PLA_2_s can be classified into two groups: i) the catalytically active enzymes, such as Asp49-PLA_2_s and ii) the catalytically inactive PLA_2_ variants (principally Lys49- and Arg49-PLA_2_s) [9]

The site of action of the venoms is marked by proteolysis, acute inflammation, clotting and bleeding. During the inflammatory response, macrophages release mediators, endothelial cells become activated and leukocytes transmigrate into the tissues [10]. The inflammatory response is modulated by the action of anti-inflammatory mediators, such as annexin A1 (AnxA1), a 37 kDa calcium and phospholipid binding protein that is an inhibitor of glucocorticoid-induced eicosanoid synthesis and PLA_2_ [11]. In addition, AnxA1 induces shedding of L-selectin in tissues which inhibits the adhesion, migration and recruitment of neutrophils to the inflamed site and accelerates neutrophil apoptosis [12, 13]. These anti-inflammatory effects of AnxA1 represent a key role in the modulation of the inflammatory response.

In spite of the evidence indicating that AnxA1 modulates pathologic processes related to inflammation, the use of either anti-inflammatory or other associated drugs along with antivenom is not widespread in Brazil [14]. The conventional antivenom treatment is inefficient in the prevention of local effects; however, some anti-inflammatory drugs have been shown to significantly reduce the tissue damage provoked by bothropic envenomation [15]. Therefore, investigations of complementary therapies are warranted to understand the inflammatory cellular mechanisms and develop novel, alternative therapeutic strategies.

The aim of this study was to analyse the effects of Ac2-26 post-treatment on the local and systemic effects of *B*. *moojeni* crude venom (CV) and the purified catalytically inactive Lys-49 PLA_2_ (MjTX-II) [16, 17]. Thus, we studied the action of these molecules on the recruitment of neutrophils, and the activation of macrophages and mast cells in the mesentery. The histopathology and the transmigration of macrophages were analyzed in the kidneys and used as indicators of systemic effects. Considering the important role of AnxA1 in the inflammatory response, the endogenous expression of this protein in the mesenteric inflammatory cells as well as in the renal structures was also evaluated.

## Materials and Methods

### Ethics statement and animals

Male Wistar rats weighing 150–200 g were randomly distributed into nine groups (n = 5/group) and were housed with a 12 h light–dark cycle and allowed food and water *ad libitum*. The experiments were performed in strict accordance with the Brazilian laws of protection and this study was approved by the Committee on the Ethics of Animal Experiments of the São José do Rio Preto Medical School, São Paulo, Brazil (Permit Number: 5314/2009).

The crude lyophilized *B*. *moojeni* venom (CV; 250 mg) was purchased from the Sanmaru Serpentarium (SANMARU Ltda., Taquaral, São Paulo, Brazil) and the MjTX-II was purified following the published procedure [16].

### Model of Inflammation

The experimental peritonitis was induced by intraperitoneal (i.p.) injection of 100 μg CV or 100 μg of MjTX-II, diluted in 0.5 mL PBS. MjTX-II constitutes approximately 7% of the crude venom of *B*. *moojeni* [16]. Control rats were injected with 0.5 mL PBS (i.p.). The animals were anesthetized with isoflurane (1%) before the experimental treatments were performed and were sacrificed either 4 or 24 hours later by an overdose of ketamine and xylazine anesthetic (100 and 20 mg/kg; i. p.).

### Pharmacological treatment with N-terminal peptide of AnxA1 (Ac2-26)

The rats were treated with the N-terminal peptide Ac2-26 (Ac-AMVSEFLKQAWFIENEEQEYVQTVK; Invitrogen, USA), 1 mg/kg (i.p.), 15 minutes after peritonitis induction by either CV or MjTX-II to determine the therapeutic efficacy of the AnxA1.

### Histopathological analyses

The kidney and mesenteric samples were fixed in 4% buffered formalin for 24 hours, dehydrated in graded ethanol and embedded in paraffin for immunohistochemical and histopathological analysis.

*Kidneys*: Sections of 4 μm were stained with hematoxylin–eosin and analyzed using a high-power objective (40X) on an Axioskop 2-Mot Plus Zeiss microscope (Carl Zeiss, Jena, Germany).

*Mesentery*: The mesenteric fragments were also fixed in 4% paraformaldehyde and 0.5% glutaraldehyde, sodium cacodylate buffer 0.1 M (pH 7.4) for 24 h at 4°C, dehydrated by graded methanol, and embedded in LR Gold resin (London Resin; Reading, Berkshire, U.K.) for the quantitative analysis of the neutrophils and mast cells. The mesenteric infiltrating cells were evaluated in 1 μm tissue sections embedded in LRGold and counted using a high-power objective (40x) on an Axioskop 2-Mot Plus Zeiss microscope (Carl Zeiss, Jena, Germany). The average number of neutrophils and mast cells was calculated and recorded. The values were reported as the mean ±SEM of the number of cells/mm^2^. Mast cells were also analyzed morphologically and classified as either intact or degranulated after staining with toluidine blue 0.5% (Taab Laboratories, UK).

### Quantitative analysis of blood and peritoneal neutrophils

The peritoneal exudates and the blood obtained by cardiac puncture were diluted 1:10 in Turk’s solution (0.1% crystal violet in 3% acetic acid) for neutrophil quantification in a Neubauer chamber (Laboroptik; Friedrichsdorf, Hessen, Germany). The values are reported as the mean ± SEM of the number of cells x 10^5^/mL.

### Immunohistochemistry analysis

The mesenteric and kidney paraffin-embedded sections (4 μm) were submitted to an antigen-retrieval step using a citrate buffer (pH 6.0) at 96°C for 30 min. The endogenous peroxide activity was blocked with 3% hydrogen peroxide for 30 min followed by an incubation overnight at 4°C with either polyclonal rabbit anti-AnxA1 (Zymed Laboratories, Cambridge, UK) or monoclonal mouse anti-rabbit anti-ED-1 (Serotec, Oxford, UK) (1:2000 and 1:500, respectively, in 1% BSA diluted in PBS) antibodies. Some sections were incubated with 1% BSA instead of the primary antibody to provide a negative control for the reaction. After washing, the sections were incubated with a secondary biotinylated Ab (Dako, Cambridge, UK). Positive staining was detected using a peroxidase-conjugated streptavidin complex and the color was developed using the DAB substrate (Dako, Carpinteria, USA). Finally, the sections were washed in distilled water, counterstained with hematoxylin, and mounted. The analysis was conducted on an Axioskop 2-Mot Plus Microscope (Carl Zeiss, Jena, GR), using the AxioVision software for densitometric and quantitative analysis.

The densitometric analysis were conducted to determine the amount of AnxA1 in the mesenteric neutrophils and mast cells (objective x63) and the juxtamedullary nephrons (objective x20) on an arbitrary scale from 0 to 255, and the data were expressed as the mean ± (±SEM). The quantification of macrophages is reported as the mean ± SEM of the number of cells/mm^2^ in mesentery and the cells/20 fields (using a high-power objective x40) in the renal juxtamedullar region.

### Cytokine levels

Aliquots of peritoneal exudate were centrifuged at 400 x g for 10 min and tested for transforming growth factor IL-1β and IL-6 by ELISA. The cytokine concentrations were measured using a commercially available immunoassay kit (R&D Systems, Minneapolis, MN, USA) and the levels were estimated according to the manufacturer’s instructions. All experiments were performed in duplicate and the data were expressed as the means ± SEM.

### Statistical analysis

The data were analyzed using GraphPad software version 4.00. Statistical differences between means were determined by analysis of variance followed, if significant, by the Bonferroni test. A probability value less than 0.05 was considered to be significant.

## Results

### Post-treatment with the Ac2-26 peptide downregulates the inflammatory response induced by the administration of CV and MjTX-II

**Fig 1:**
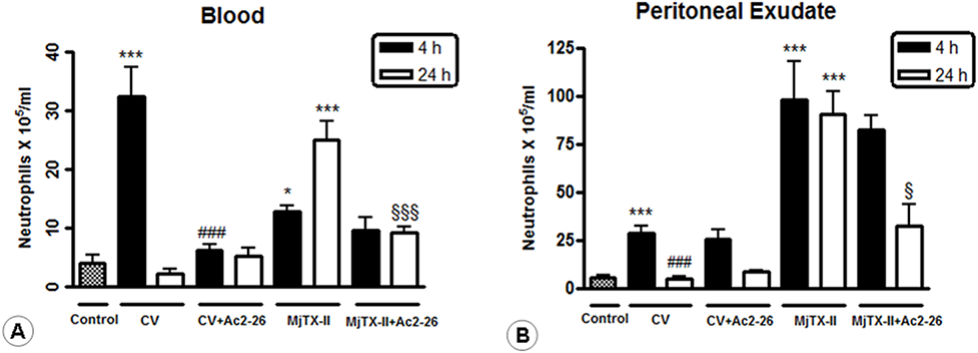
Blood and peritoneal neutrophils after CV- and MjTX-II-induced peritonitis. Blood (A) and peritoneal (B) neutrophil counts. Peritonitis was induced in rats by i.p. injection of either CV or MjTX-II (100 μg) in 0.5 mL of PBS. Control animals were injected with PBS alone. Another set of animals from CV and MjTX-II groups were treated i.p. with 1 mg/kg of Ac2-26 peptide 15 minutes after the induction of peritonitis. The data represent the mean ± SEM of the cell numbers x 10^5^ per mL. *P < 0.05 and ***P < 0.001 vs control; ^###^P < 0.001 vs CV 4h; ^§^P < 0.05 and ^§§§^P < 0.001 vs MjTX-II 24h.

Initially, we examined the inflammatory response profile resulting from CV- and MjTX-II-induced peritonitis. In the initial phase of peritonitis (4 hours) CV markedly increased the influx of blood neutrophils in comparison to the control groups. MjTX-II administration produced a similar effect only at 24 hours (Fig 1A). Consistent with these results, the peritoneal exudates from animals treated with CV (4 hours) and MjTX-II (4 and 24 hours) also exhibited a significant increase in the number of neutrophils, (Fig 1B) when compared with the control animals.

Ac2-26 post-treatment efficiently reduced the blood neutrophil influx provoked by CV and MjTX-II-induced peritonitis (Fig 1A) at 4 and 24 hours, respectively. This effect was associated with diminished peritoneal neutrophil recruitment in the MjTX-II group at 24 hours, but no significant effect was observed in the CV group (Fig 1B).

**Fig 2:**
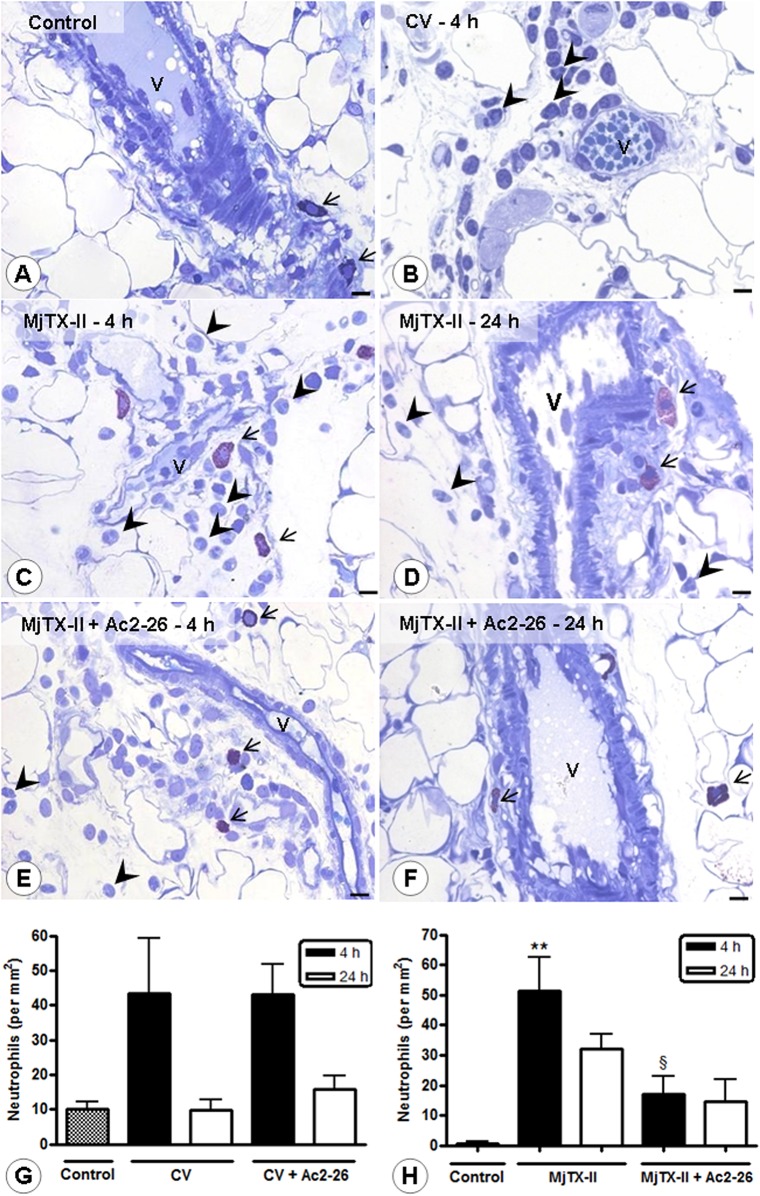
Effect of Ac2-26 treatment on the mesenteric inflammation induced by CV and MjTX-II. Intact mast cells (arrows) in the control mesentery (A). Inflamed mesentery of CV—(B) and MjTX-II-induced peritonitis (C-D) with extravasated neutrophils in the tissue (arrowheads) as observed at 4 and 24 h. Reduced neutrophil influx (arrowheads) after Ac2-26 post-treatment at 4 (E) and 24 h (F) of MjTX-II-induced peritonitis. Vessels (V). Stain: Toluidine blue. Scale bars: 10 μm. Quantitative analysis of extravasated neutrophils in the mesentery after CV- (G) and MjTX-II–induced peritonitis (H). The data represent the mean ± SEM of cell numbers/mm^2^ (n = 5 animals/group). **P < 0.01 vs control; ^§^P < 0.05 vs MjTX-II-4 h.

In addition, the peritoneal exudates displayed a marked increase in the IL-1β and IL-6 levels 4 hours after CV and MjTX-II administration (Table 1). As expected, the Ac2-26 treatment efficiently reduced the levels of both proinflammatory cytokine levels, although only IL-6 was significantly different between untreated and Ac2-26-treated CV group (Table 1). Similarly, an inflammatory response was detected in the mesenteries 4 hours after CV and MjTX-II injection and was characterized by elevated numbers of extravasated neutrophils and mast cell activation compared to the control animals (Fig 2A–2C, 2G and 2H; Table 2). Additionally, CV triggered a significant increase in the number of macrophages in the mesentery (Table 2) whereas no change was observed in the number of macrophages following MjTX-II administration. At 24 h of peritonitis, no alterations were observed in the inflammatory cell counts (Fig 2D, 2G and 2H; Table 2).

**Table 1:** Effect of Ac2-26 treatment on proinflammatory cytokine secretion.

Time (h)/ Treatment	IL-1 (pg/mL)	IL-6 (pg/mL)
0 (control)	82 ± 24	ND
4 / CV	612 ± 186 *	1199 ± 383 ***
24 / CV	44.5 ± 7	ND
4 / CV + Ac2-26	293 ± 54	278 ± 64 ##
24 / CV + Ac2-26	72.5 ± 16	ND
4 / MjTX-II	1086 ± 295.5 ***	959 ± 245 ***
24 / MjTX-II	58 ± 14	ND
4 / MjTX-II + Ac2-26	570 ± 122	456.5 ± 90
24 / MjTX-II + Ac2-26	37 ± 16	ND

Peritonitis was induced in rats by i.p. injection of CV (100 μg) or MjTX-II (100 μg) in 0.5 mL of PBS. Control group received i.p. only PBS. Another set of animals were treated i.p. with 1 mg/kg of Ac2-26 peptide after 15 minutes of CV or MjTX-II injection. Peritoneal exudate was collected 4 and 24 hours after peritonitis inductions and cytokine dosages were performed by ELISA, as described in Methods. Values are expressed as the mean ± SEM of cytokine levels (n = 5 rats/group). ND < 31.25 pg/mL (not detected).

* P < 0.05 and

*** P < 0.001 vs control

^##^P < 0.01 vs CV 4 h.

**Table 2:** Quantitative analysis of degranulated mast cell and macrophages into mesentery.

Time (h)/ Treatment	Degranulated mast cell (% of cells/mm^2^)	Macrophages (cells/mm^2^)
0 (control)	25 ± 15	45.3 ± 13.3
4 / CV	64.5 ± 12.2**	133 ± 10***
24 / CV	46 ± 13.8	60.3 ± 2.8
4 / CV + Ac2-26	33.1 ± 11^#^	29.5 ± 4.9^###^
24 / CV + Ac2-26	25.4 ± 12.3	46.7 ± 3
4 / MjTX-II	62,2 ± 11,7**	56 ± 10.6
24 / MjTX-II	48 ± 6,4	30.6 ± 7.3
4 / MjTX-II + Ac2-26	39,6 ± 12	61 ± 10.1
24 / MjTX-II + Ac2-26	31,4 ± 17	39.8 ± 12.7

Peritonitis was induced in rats by i.p. injection of CV (100 μg) or MjTX-II (100 μg) in 0.5 mL of PBS. Control group received i.p. only PBS. Another set of animals were treated i.p. with 1 mg/kg of Ac2-26 peptide after 15 minutes of CV or MjTX-II injection. Mesentery was collected after 4 and 24 hours after peritonitis induction and cellular counts performed as described in Methods. Values are expressed as the mean ± SEM of the number of cells per mm^2^ (n = 5 rats/group).

** P < 0.01 and

*** P < 0.001 vs control

^#^P < 0.05 and

^###^P < 0.001 vs CV 4 h.

Post-pharmacological treatment with Ac2-26 was effective in inhibiting the influx of neutrophils into the mesentery in the peritonitis induced by MjTX-II, but not by CV (Fig 2E–2H). The anti-inflammatory effects of the peptide were associated with mast cell activation in CV and MjTX-II-peritonitis, and macrophage recruitment to the mesentery that presented diminished cell counts in the CV-induced peritonitis (Table 2).

### Endogenous AnxA1 expression is modulated in inflammatory cells by the administration of CV and MjTX-II

**Fig 3:**
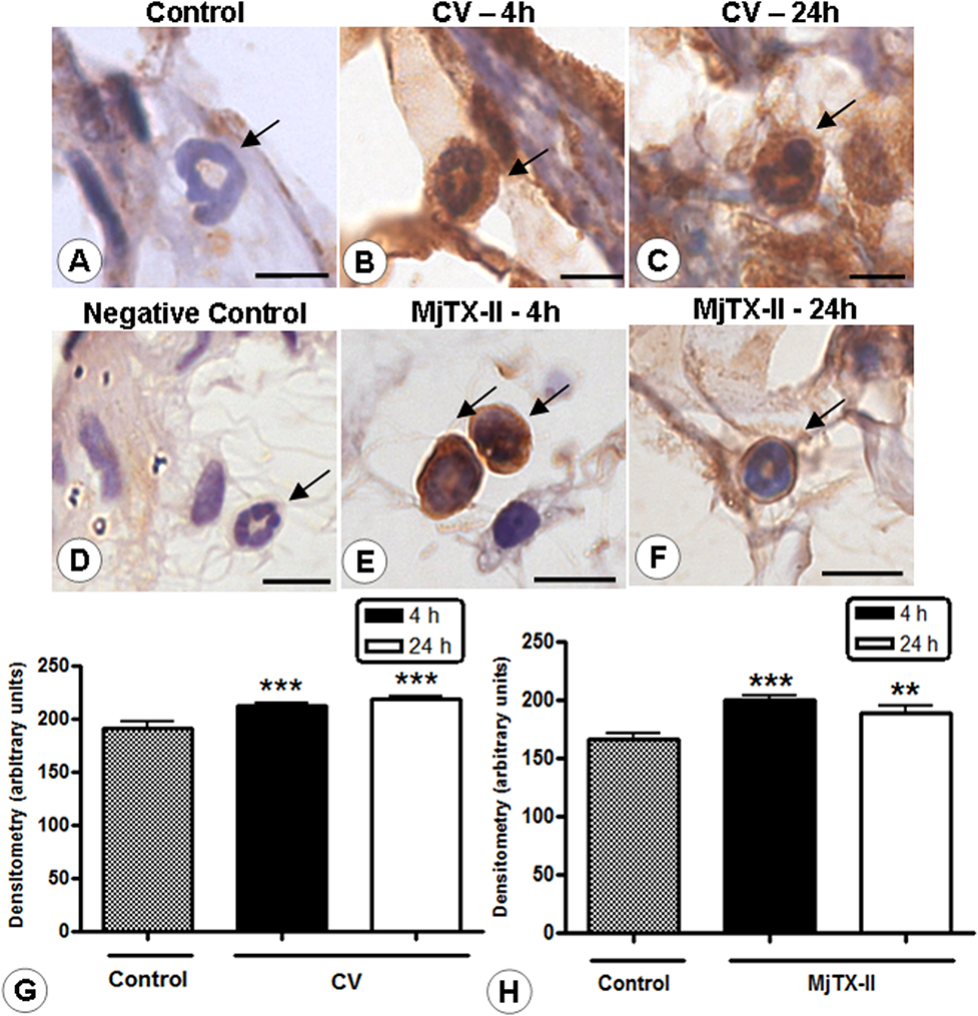
Expression of AnxA1 in the mesenteric neutrophils. Strong AnxA1 immunoreactivity in neutrophils (arrows) from CV (B, C) and MjTX-II (E, F) groups, after 4 and 24 h, compared with cells of control group (A). The absence of immunoreactivity in the negative control (D). Scale bars: 5 μm. Counterstain: hematoxylin. Densitometric analysis of mesenteric neutrophils immunostained for AnxA1 in the CV (G) and MjTX-II (H) groups. The values (arbitrary units) are expressed as mean ± SEM of the sections analyzed from 5 rats /group. **P < 0.01 and ***P < 0.001 vs control.

After 4 and 24 hours of either CV or MjTX-II administration, we observed extravasated neutrophils and intense AnxA1 immunoreactivity compared to the control group (Fig 3A–3C, 3E and 3F). We did not detected immunostaining in the cells used as a negative control (Fig 3D). These histological observations were confirmed by densitometric analysis of AnxA1 (Fig 3G and 3H).

**Fig 4:**
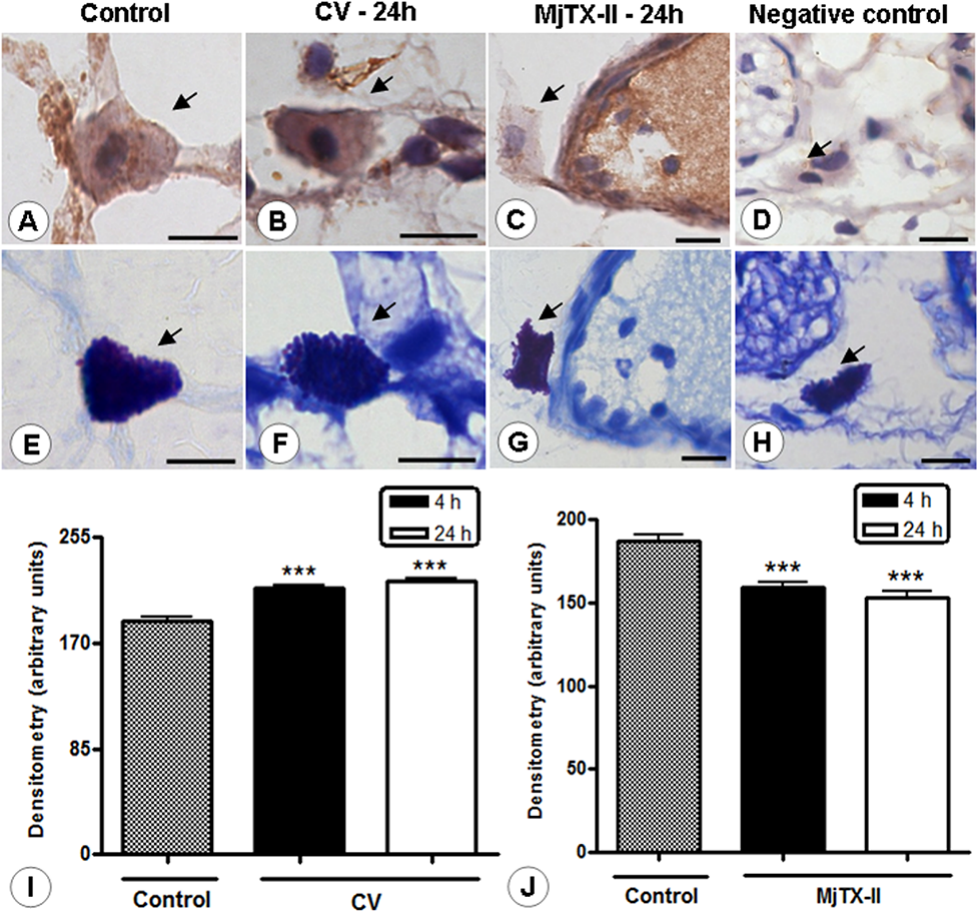
Light micrographs of rat mesenteries showing AnxA1 immunoreactivity in the mast cells. AnxA1-positive mast cells in the control group (A). After 24 hours, the administration of CV and MjTX-II was associated with intense (B) and low (C) AnxA1 immunoreactivity in mast cells (arrows), respectively. Negative control of reaction (D). Counterstain: hematoxylin. Stained adjacent histological sections with 0.5% toluidine blue confirms that the indicated cells are mast cells (arrows; E-H). Scale bars: 5 μm. Densitometric analysis of mesenteric mast cells immunostained for AnxA1 in CV (I) and MjTX-II (J) groups. The values (arbitrary units) are expressed as the mean ± SEM of sections analyzed from 5 rats /group. ***P < 0.001 vs control.

Similar results were obtained for mesenteric mast cells in CV-induced peritonitis, and we detected high endogenous levels of AnxA1 in these cells at 4 and 24 hours (Fig 4A and 4B). Following MjTX-II administration, the AnxA1 expression was significantly decreased in mast cells (Fig 4C). Fig 4D presents the section incubated in the absence of the primary antibody to provide a negative control of the reaction. The histological sections, sequential to those obtained for immunohistochemistry (Fig 4E–4H), were stained with toluidine blue (Fig 3F) to confirm that the immunostained cells were mast cells. The densitometric analysis of AnxA1 expression on mast cells corroborates our histological findings (Fig 4I and 4J).

### Ac2-26 restores the renal damage induced by the administration of CV and MjTX-II

**Fig 5:**
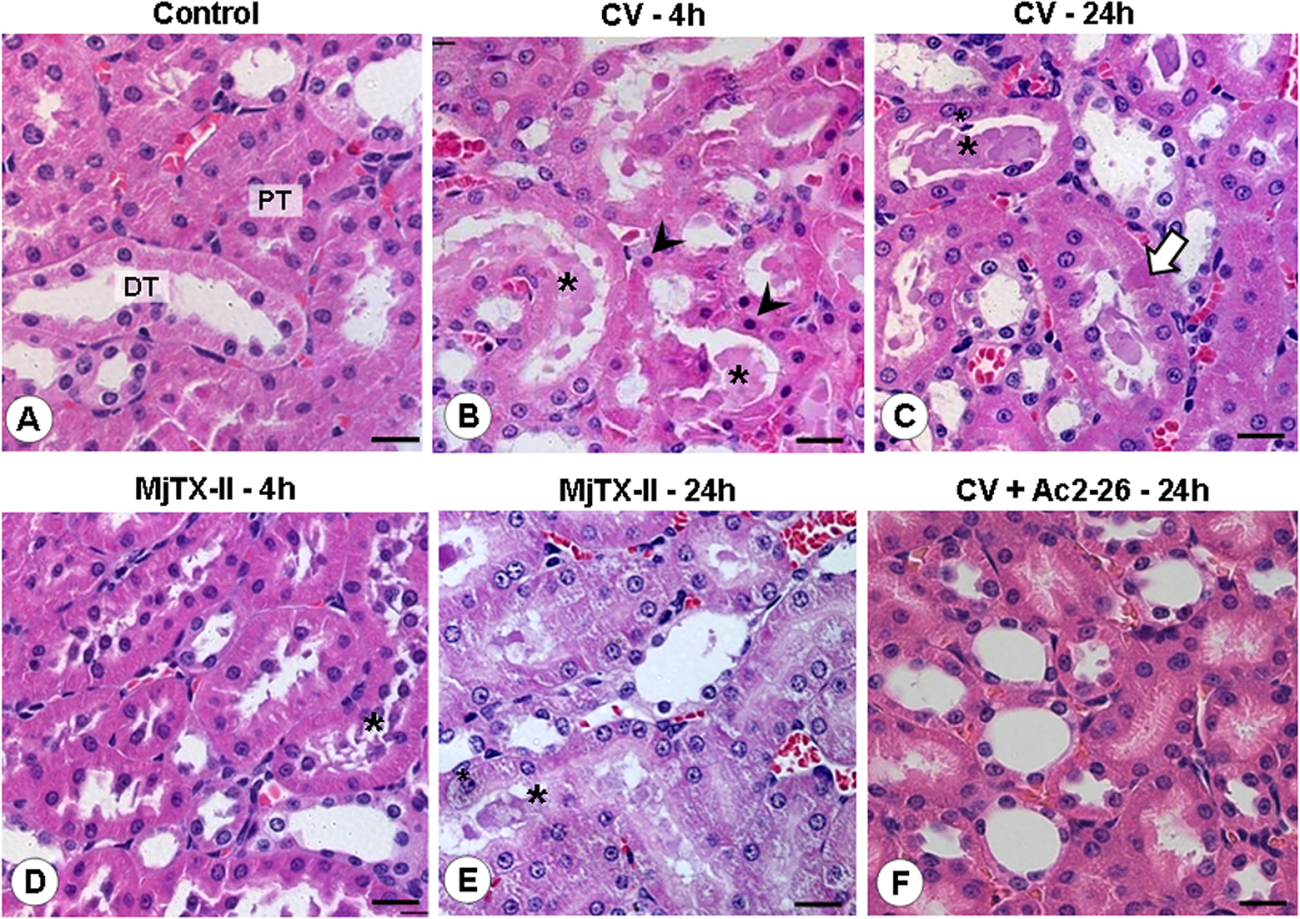
Histopathological analysis of kidneys after CV- and MjTX-II-induced peritonitis. (A) Normal histological condition of proximal (PT) and distal convuluted tubules (DT) in the renal control. (B-E) CV- and MjTX-II PLA_2_-induced peritonitis provoked renal damage characterized by the presence of pyknotic nuclei (arrowheads), karyolisis (white arrow) and hyaline casts (asterisks) in proximal tubules. (F) Ac2-26 peptide restores the normal morphological aspect of juxtamedullary structures. Stain: Hematoxylin-Eosin. Scale bars: 20 μm.

After the characterization of the local effects of CV and MjTX-II administration, we evaluated the systemic damage. For this evaluation, the kidneys were examined 4 and 24 hours after CV and MjTX-II–induced peritonitis. As expected, morphological alterations were detected specially in the proximal (PT) and distal (DT) tubules of juxtamedullary nephrons in the treated group compared to the control group (Fig 5). At 4 hours, the CV group exhibited renal tubular epithelial cells with conspicuous and pyknotic nucleus (Fig 5B) and, at 24 hours, some epithelial cells from the proximal tubules lacked nuclei (Fig 5C), which indicates a process of necrosis and karyolisis. Additionally, hyaline casts, produced by detachment of epithelial cells, were detected in the renal tubular lumens in both CV and MjTX-II groups and at both time points (Fig 5B–5E). Post pharmacological treatment with Ac2-26 promoted significant recovery of juxtamedullary structures in the CV (Fig 5F) and MjTX-II groups.

**Fig 6:**
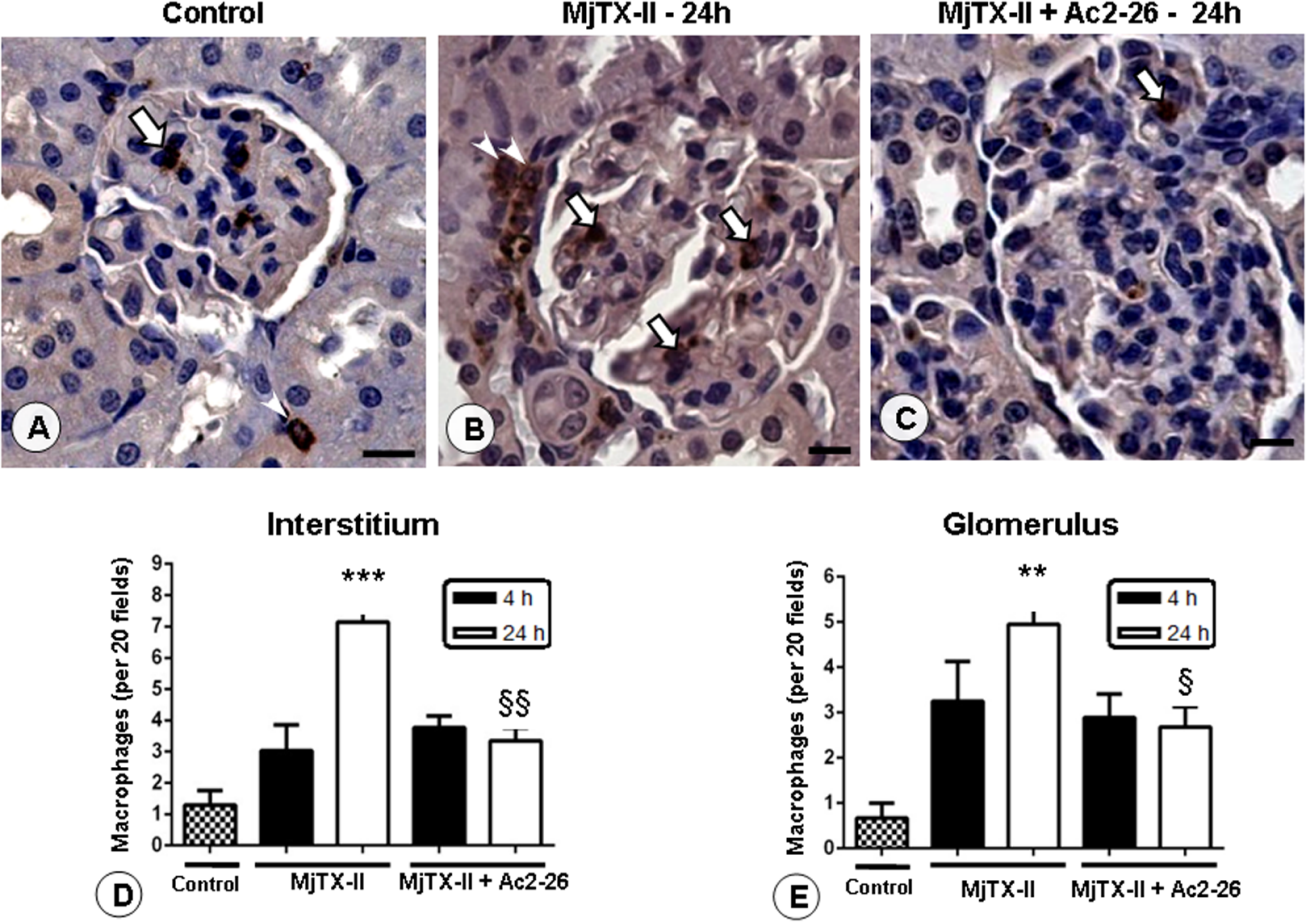
Presence of renal macrophages after MjTX-II-induced peritonitis. An increased number of cells was observed in the glomeruli (arrows) and interstice (arrowheads) of the juxtamedullary region 24 h after peritonitis induced by MjTX-II (B), compared to the control group (A). Ac2-26 peptide post-treatment prevented the macrophages influx in renal tissue (C). Scale bars: 20 μm. Counterstain: Hematoxylin. The data represent the mean ± SEM of the number of interstitial (D) and glomerular (E) macrophages per 20 fields in the renal tissue (n = 5 animals/group). **P < 0.01 and ***P < 0.001 vs control; ^§^P < 0.05 and ^§§^P < 0.01 vs MjTX-II 24 h.

In addition to the morphological alterations, we observed that the administration of MjTX-II caused a marked influx of macrophages into the renal glomerulus and interstitium (Fig 6) at 24 hours, as detected by anti-ED-1 antibodies. The treatment with Ac2-26 peptide efficiently decreased the numbers of macrophages in both renal compartments in the MjTX-II group at 24 hours (Fig 6C–6E), but not in the CV group, suggesting an inhibitory regulation of monocyte recruitment into the tissue.

### CV and MjTX-II upregulates AnxA1 expression in renal tissues

**Fig 7:**
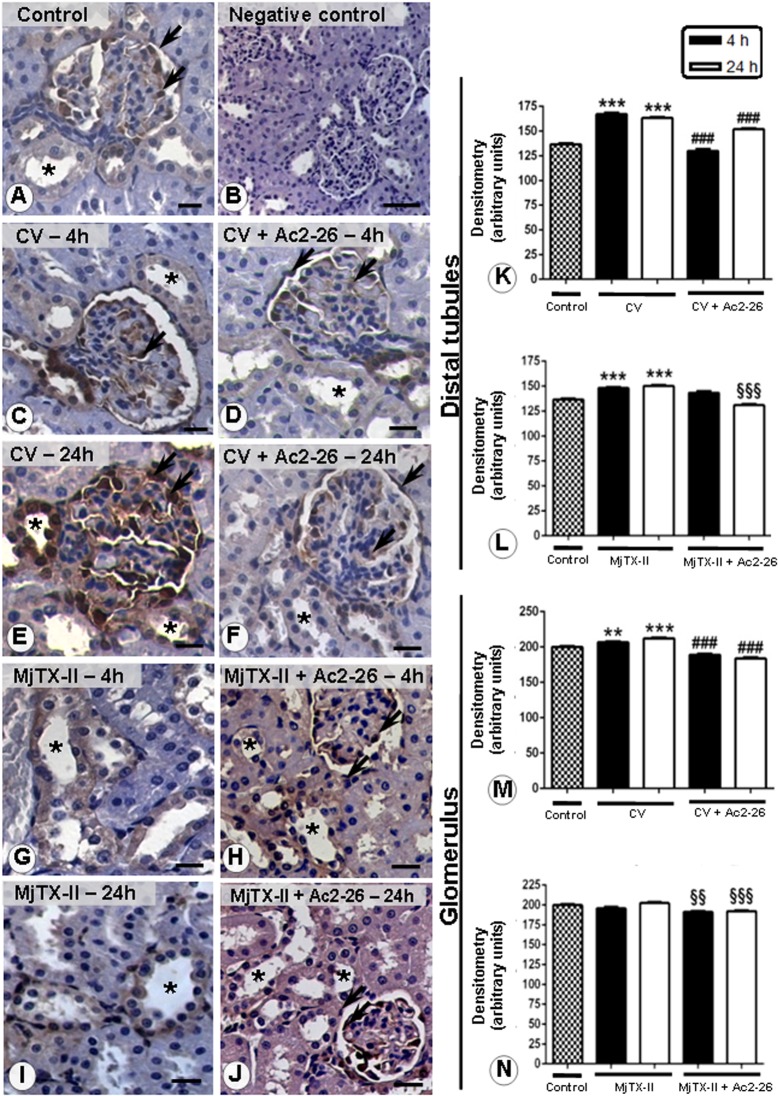
Analysis of AnxA1 expression in the juxtamedullary renal structures. AnxA1 immunostaining was detected in the epithelial cells under all of the experimental conditions (A, C, E, G, I), concentrated in the glomerular capsule (arrows) and distal convuluted tubules (asterisks). Ac2-26 post-treatment decreased the immunostaining for endogenous AnxA1 in the distal tubules and glomerular capsule (D, F, H, J) 4 and 24 h after CV- and MjTX-II-induced peritonitis. Negative control of the reaction (B). Counterstain: hematoxylin. Scale bars: 20 μm. Densitometric analysis of the epithelial cells from distal convuluted tubules (K-L) and glomerular capsule (M-N) immunostained for AnxA1. Values (arbitrary units) are expressed as the mean ± SEM of sections analyzed from 5 rats /group. **P < 0.01 and ***P < 0.001 vs control; ^###^P < 0.001 vs CV groups; ^§§^P < 0.01 and ^§§§^P < 0.001 vs MjTX-II groups at the corresponding experimental time.

Endogenous AnxA1 immunostaining was analyzed in the juxtamedullary nephrons in all experimental groups and its expression was detected in epithelial cells, especially from the glomerular capsule and distal convoluted tubules (Fig 7).

CV administration induced increased levels of AnxA1 in the epithelial cells from both compartments (distal tubules and glomerular capsules) after 4 and 24 hours, compared to the control group (Fig 7A–7C). Similar observations were made in the epithelial cells from distal tubules of MjTX-II group at both time points (Fig 7D and 7E), but no alterations were detected in the renal capsule. Absence of immunoreactivity was observed in the negative control (Fig 7F). Post-treatment with Ac2-26 peptide decreased immunostaining for endogenous AnxA1 in epithelial cells from the distal tubules and renal capsule 4 and 24 h after CV and MjTX-II administration (Fig 7G–7J). The densitometric analysis confirmed our histological findings (Fig 7K–7N).

## Discussion

The anti-inflammatory effects of AnxA1 and its mimetic peptide Ac2-26 have been reported in several *in vivo* models of acute, chronic and systemic inflammation [18–21]. However, few studies have monitored their pharmacological actions in local and systemic inflammatory processes caused by ophidic accidents. We examined the protective effect of Ac2-26 post pharmacological treatment on local and systemic inflammatory processes induced by intraperitoneal injection of *B*. *moojeni* crude venom (CV) and its Lys-49 phospholipase A_2_ (MjTX-II) in rats.

CV-induced peritonitis caused an intense inflammatory response at 4 hours characterized by increased numbers of blood, peritoneal and mesenteric neutrophils, which was absent at 24 hours. Our findings are in agreement with rodent models of carrageenan and zymosan peritonitis [11, 18] which are characterized by the high number of neutrophils that are circulating and transmigrating to the peritoneal cavity in the first 4–6 hours. Previous investigations with *Bothrops* venoms also observed leukocyte infiltration, predominantly composed of neutrophils, at the site of injury in the first hours after venom application [22, 23]. Additionally, *B*. *asper* venom stimulates the expression of adhesion molecules, such as L-selectin, LFA-1, ICAM-1, PECAM-1 and CD-18 on neutrophils and endothelial cells which facilitates their transmigration into the injured tissue [24].

MjTX-II administration also induced neutrophilia at 4 (blood, peritoneal exudates and mesentery) and 24 hours (peritoneal exudates and blood), demonstrating that the bothropic venom myotoxin participates in neutrophil transmigration into the tissue. These findings corroborate the data that in the gastrocnemius muscle of animals that received *B*. *moojeni* PLA_2_, leukocyte infiltration and drastic myonecrosis was observed after 24 hours [25]. We have no direct evidence that MjTX-II can stimulate the bone marrow; however, the rapid increase of inflammatory cells in blood and peritoneal fluids that we observed may indicate that this PLA_2_ stimulates bone marrow proliferation. This effect has been suggested by models of peritonitis induced by a metalloproteinase from *B*. *asper* [26] and a toxin from *B*. *jararaca* [27], in which the production of myeloblasts, myelocytes and granulocytes precursor cells was increased.

The increased numbers of degranulated mast cells in the mesentery 4 hours after induced peritonitis are also supported by other studies using *B*. *jararaca* and *Protobothrops mucrosquamatus* crude venoms, as well as PLAs_2_ isolated from *B*. *jararacussu* and *B*. *atrox* [28–31]. The importance of mast cells in neutrophil migration was demonstrated in peritonitis and uveitis models, where mast cells were selectively depleted using 48/80 compound [32, 33]. These experiments indicated a reduction in neutrophil influx into the peritoneal cavity after 4 hours of zymosan-induced peritonitis [32] and to the aqueous humor after 24 hours of lipopolysaccharide-induced uveitis [33] compared to animals whose cells were not depleted. The potent mediators released by mast cell degranulation, especially prostaglandin 2 (PGD2), histamine and leukotrienes, are responsible for the local inflammatory process and edema induced by *B*. *moojeni* venom [34]. The amines released by mast cells and phospholipids hydrolysis may act as possible mechanisms to the edema induction by *B*. *pirajai* MjTX-II [35]. On the other hand, mast cell activation by snake venoms leads to the release of carboxypeptidases A and other proteases, that can degrade the venom components [36].

The involvement of PLA_2_ in edema has been described in several studies, but this mechanisms remains unclear [37, 38]. Both the catalytic activity [25] and the positively charged content [35, 39] present in the majority of secreted PLA_2_ have been described in several reports and are considered to be important for its inflammatory activity. A possible mechanism to explain the different pharmacological effects of the catalytically inactive PLA_2_s from snake venoms, such as Lys-49 (MjTX-II), is based on the hydrophobic cationic stretch located in the C-terminal region, which permits the inactive PLA_2_s to embed in and disorganize the membrane [30, 35, 39–41].

Ac2-26 post-treatment markedly reduced the proportion of degranulated mast cells in the mesenteries of treated rats at 4 hours in both models of peritonitis (CV and MjTX-II) as well as the circulating neutrophils after 4 hours of CV (~ 81%) and after 24 hours of MjTX-II (~63%). For MjTX-II-induced peritonitis, the peptide treatment was more effective in modulating neutrophil recruitment, as shown by the significant reduction of mesenteric neutrophils after 4 hours (~ 65%), as well as that of circulating (~63%) and peritoneal cells (~65%) at 24 hours, compared to untreated rats. Additionally, the anti-inflammatory effect of Ac2-26 was also associated with the inhibition of proinflammatory cytokine release, IL-1β and IL-6, 4 hours after the administration of both CV and MjTX-II. Our findings support the anti-inflammatory roles of AnxA1 and its mimetic peptide described in the literature under different inflammatory conditions and associated with neutrophil transmigration [20, 42–45] cytokine levels [20, 46], and mast cell activation [42, 47].

Mast cells are promising candidates to be activated by PLA_2_s from snake venoms, and, therefore, to release vasoactive inflammatory mediators such as histamine [30, 35, 48, 49]. Compounds rich in cationic charges such as Lys-49 MjTX-II activate rat mast cells in vitro [50, 51] and in vivo [52]. Because plasma membranes from mast cells contain fixed anionic sites constituted mainly of sulfated glycosaminoglycans [48, 53], it is proposed that mast cell activation by the cations of MjTX-II takes place by electrostatic interactions with these cell surface anionic sites [30]. Thus, Ac2-26 contributed to the reduction of mast cell activation by MjTX-II, thereby inhibiting the inflammatory cascade.

In addition to mast cells and neutrophils, macrophages are important cells with vasoactive and inflammatory properties and their study is necessary to understand the progressive tissue damage. CV- and MjTX-II-induced peritonitis promoted increased numbers of mesenteric and renal macrophages that were abrogated by pharmacological treatment with Ac2-26, demonstrating its anti-inflammatory role in moderating of tissue damage. In our laboratory, we found that Ac2-26 treatment reduced the migration of macrophages to kidney, and attenuated the hemodynamic changes and tubular injury in experimental models of cyclosporine- [43] and tacrolimus-induced nephrotoxicity [45].

Activated macrophages produce several vasoactive substances that can damage renal tissue via reactive oxygen species, nitric oxide, enzymes, and pro-inflammatory cytokines. These effects are related to mechanisms involved in CsA nephrotoxicity and we could not exclude a role for macrophages in CV and MjTX-II nephrotoxicity [54].

Renal consequences demonstrate the ability of the venom to affect remote organs, with functional alterations becoming evident only after 24 hours, coinciding with the resolution phase of local and initial systemic effects. It has been demonstrated that bothropic venom is capable of inducing renal alterations by both direct and indirect mechanisms caused by cytotoxicity and ischemia, respectively [55].

The histopathological damage observed in the kidneys suggests that acute kidney injury (AKI) [56] is responsible for the majority deaths in those that survive the initial accident symptoms [57]. This direct action has significant relevance to the features of tubular injury in AKI [58]. Tubular cells are bound together by basolateral junctions that are composed of cytoskeletal elements, and the disorganization of these junctions affects the integrity of the structure. Myotoxins present in *Bothrops* venoms destabilize the membranes of the epithelial tubular cells [59] by a mechanism which diminishes cellular permeability with respect to ions and macromolecules, resulting in calcium influx. The uncontrolled calcium entering the tubular cells activates calcium-dependent proteases and endogenous phospholipases, which compromise cytoskeletal organization. This process is considered the main cause of the casts that we observed in juxtamedullary proximal tubules after CV and MjTX-II administration, resulting in the loss of important junction and anchoring functions, leading to the detachment of these cells [60, 61]. The effects of these alterations are identical to the effects that are triggered by CV myotoxins, suggesting that the MjTX-II could be responsible for the direct action of *B*. *moojeni* CV [59, 62]. Among the induced injuries caused by CV, we also observed tubular cells with pyknotic and missing nucleious (karyolysis) that are characteristic of necrotic process. Nuclear pyknosis occurs also after renal ischemia and reperfusion [19], suggesting that the trigging of necrosis could be associated with ischemia, as an indirect effect of CV, following the induction of hemodynamic disturbances [63].

It is important to define the biological actions of Ac2-26 treatment in this inflammatory model; immunohistochemical analysis confirmed the expression of AnxA1 in the mesenteric mast cells and neutrophils as described earlier [64–66]. The administration of CV and MjTX-II triggered an increase in endogenous AnxA1 levels in neutrophils at 4 and 24 hours. In mast cells, AnxA1 expression was differently modulated by CV and MjTX-II. CV and MjTX-II were associated with high and low levels of the protein, respectively. Moreover, mast cells contain increased levels of endogenous AnxA1 in response to glucocorticoid treatment [64], chronic inflammation [67], and tumourigenesis [68]. Taken together, our results confirm that neutrophils and mast cells represent important sources of AnxA1 in the tissues and thereby contribute to the regulation of inflammatory responses triggered by CV and MjTX-II-induced peritonitis.

In the renal tissue, we provide evidence that AnxA1 expression increase in juxtamedullary distal tubules and glomeruli after CV and MjTX II administration, while post-treatment with the peptide reduced the endogenous levels of AnxA1. These results were confirmed in a model of renal ischemia/reperfusion injury [19]. Similarly, recent results from our research group demonstrated that the immunosuppressives CsA [43] and Tacrolimus [45] induce an increase in renal AnxA1 expression to protect cell function and structure [69]. In addition, renal tubular cells subject to high calcium levels *in vitro* also upregulate endogenous AnxA1, which plays a pivotal role in enhancing calcium oxalate monohydrate-cell binding and the inhibition of cell proliferation and migration [70].

**Fig 8:**
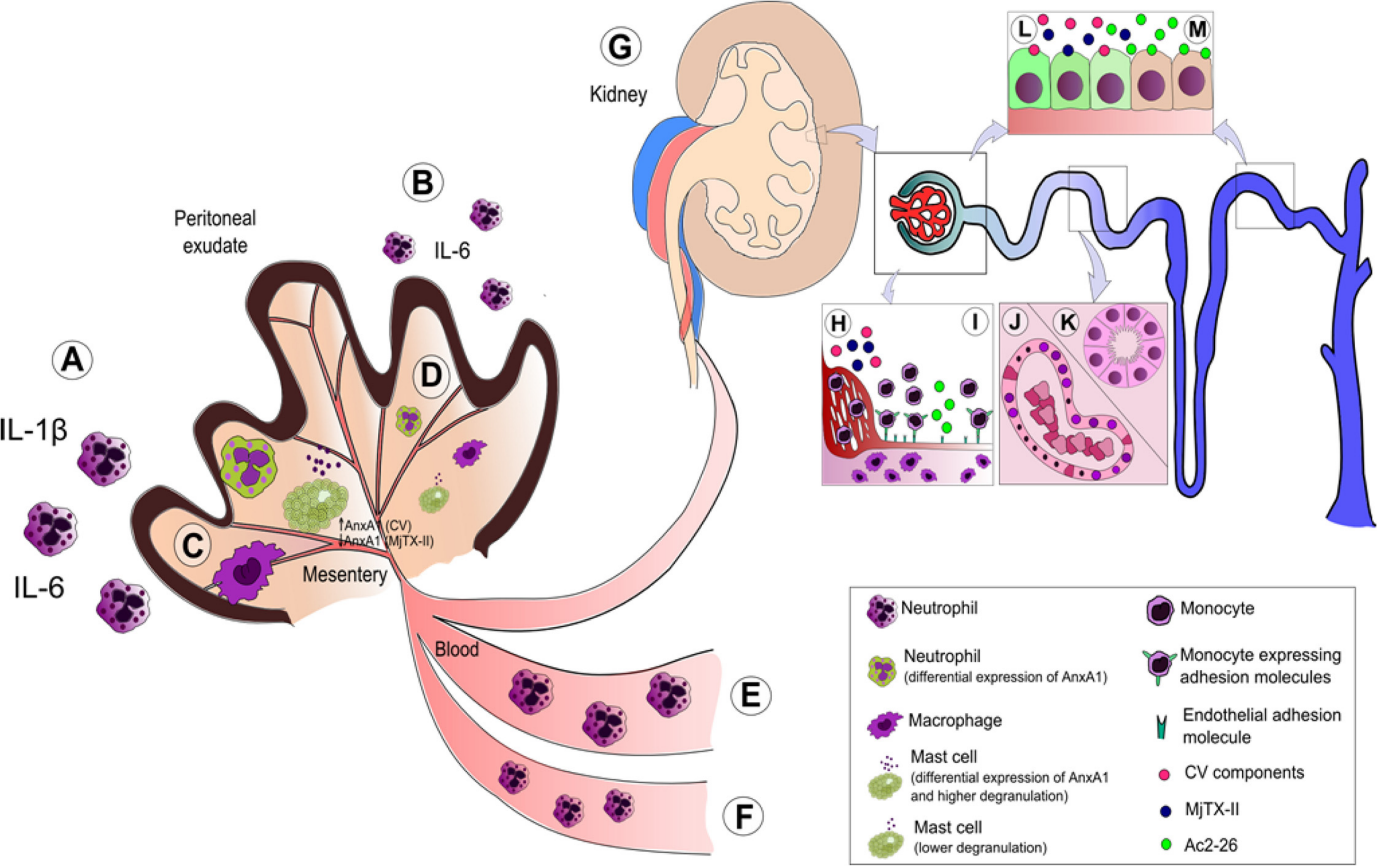
Model to summarize the effects of Ac2-26 treatment after either CV or MjTX-II induced peritonitis. Local inflammation in the peritoneal exudate augments the number of neutrophils and the pro-inflammatory cytokines, IL-1β and IL-6 (A). This inflammation is decreased after Ac2-26 treatment (B). In the mesentery, the inflammatory response promotes the influx of macrophages, and increases the number of neutrophils, degranulated mast cells and the levels of AnxA1 expression (C). The Ac2-26 treatment decreased the numbers of all the inflammatory cells (D). The systemic inflammation results in an increase of neutrophils in the bloodstream (E); these levels are restored after Ac2-26 post-treatment (F). In the kidney (G), MjTX-II augmented the infiltration by macrophages (H) and Ac2-26 prevented this influx (I). Histopathological analysis showed direct (casts) and indirect (pyknotic nuclei/karyolysis) effects of the envenoming (J), which were restored by the Ac2-26 treatment (K). Higher levels of AnxA1 were observed in the distal tubules and glomerular cells after the administration of either CV or MjTX-II (L), and these levels decreased with the anti-inflammatory treatment (M).

The findings of this study are outlined in Fig 8 and, taken together, the data provides evidence that the N-terminal peptide of AnxA1, Ac2-26, significantly attenuated the local and systemic inflammatory processes. We propose that these experimental findings may have a significant impact on and relevance for the development of new therapeutic strategies for envenomation.
